# 

**Published:** 1843-12-09

**Authors:** 


### CONTENTS.

A Case of Dissecting Aneurism of the Aorta. By E. Michener, M. D., - - - 277

Account of an Unusual Strangulation of a portion of the Large Intestines, caused by the formation of an adventitious band, between the stomach and liver. By P. Le B. Stickney, M. D., - - - - - 280

*Clinical Lectures and Reports.—Pennsylvania Hospital.*

On Hydrocele. By Dr. Randolph. Lecture 1, 281

*Philadelphia Hospital.*

On Syphilis. By Dr. Clymer. Lecture 1, - 282

*Bibliographical Notices.*

Principles of Medicine. Comprising General Pathology, Therapeutics, and a Brief General View of Etiology, Nosology, Semeiology, Diagnosis and Prognosis. By Chas. J. B. Williams, M. D., F. R. S., Professor of the Principles and Practice of Medicine, etc. etc. etc. With Notes and Additions. By Meredith Clymer, M. D., Lecturer on the Institutes of Medicine, Physician to the Philadelphia Hospital, Fellow of the College of Physicians, etc. etc. - - 286

An Experimental and Critical Inquiry into the Nature and Treatment of Wounds of the Intestines. Illustrated by Engravings. By Samuel D. Gross, M. D., Professor of Surgery in the Louisville Medical Institute, etc. etc. etc. - - - - 287

Dr. Tavernier's Lever-Belt and Busk for the Treatment of Spinal Deformities, - - 288

Dr. Bennett on Inflammation of the Nervous Centres, - - - - - - - 288

